# Supplementary material for: Aging effects in Schelling segregation model
Source: Sci Rep. 2022 Nov 12;12:19376. doi: 10.1038/s41598-022-23224-7 (PMC9653388; doi:10.1038/s41598-022-23224-7)
Supplement: Supplementary file 1 — Supplementary Information 1. [file 41598_2022_23224_MOESM1_ESM.pdf]

Supplementary Information for "Aging effects in Schelling Segregation Model"

David Abella[1*], Maxi San Miguel[1], and José J. Ramasco[1]

[1]Instituto de Física Interdisciplinar y Sistemas Complejos IFISC (CSIC-UIB), Campus UIB, 07122 Palma de Mallorca, Spain
[*]david@ifisc.uib-csic.es

S3. Effect of a higher vacancy density ρ_v in the coarsening dynamics

Since we restrain ourselves to the region $\rho_v < 0.5$, the increase/decrease of the number of vacancies does not change dramatically the behaviour. Above this value, we approach the segregated-dilute transition ($\rho_v \sim 0.62$). Nevertheless, it is worth to mention a few features we observe on the coarsening dynamics.

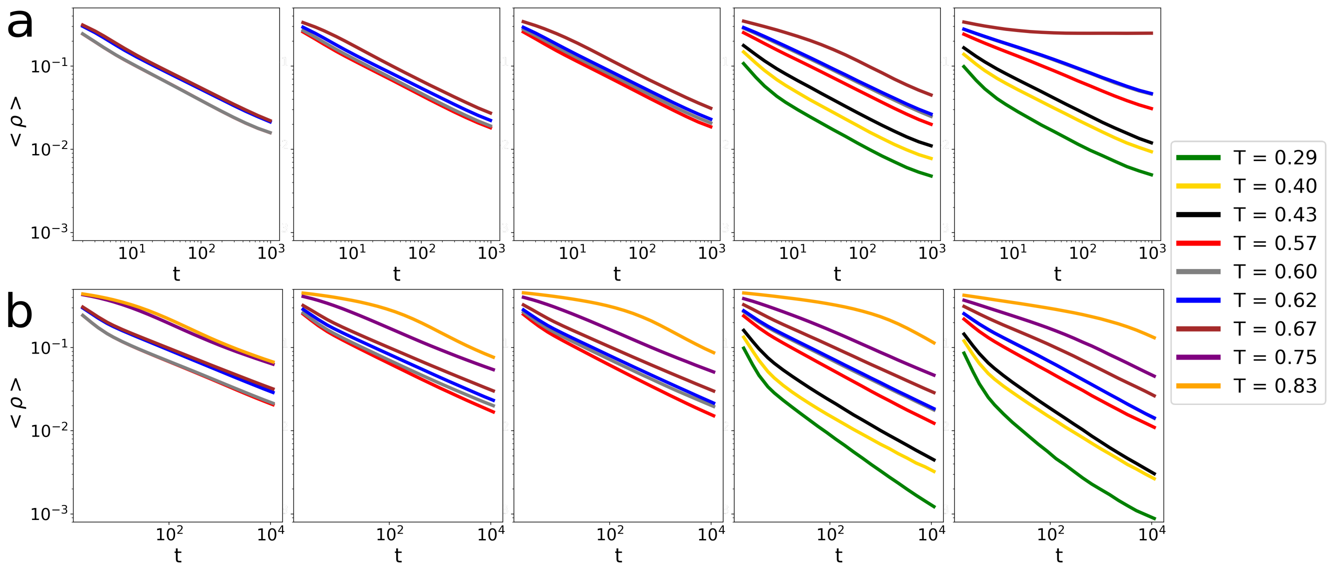

Figure 1. Average interface density $\langle \rho(t) \rangle$ as a function of time steps for different values of the tolerance parameter T fr the Schelling model (**a**) and the version with aging (**b**). The different plots show a the evolution at a different value of the vacancy density, increasing from left to right $\rho_v = 0.005$, 0.15, 0.2, 0.3 and 0.45. Average performed over 10^3 realisations with system size 100 x 100.

Essentially, when we set a higher vacancy density, the number of agents which see vacancies at their surroundings increases. This results in a family of similar power-law decays towards the segregated state for every meaningful value of T (see Fig. 1).

Moreover, a higher ρ_v allows us to study the coarsening phenomena for lower values of T according to the phase diagram for the original Schelling model. For those particular cases, when the aging is introduced, we observe a power law decay faster than without aging (Fig. 1b). Therefore, the aging effect accelerates segregation in this region of the phase diagram, contrary as for lower values of ρ_v. This acceleration is not caused by reaching the 2-clusters state in less time. Since there is a large presence of vacancies, aging causes a formation of vacancy clusters at the interface. Fig. 2 shows the final segregated state with and without aging. This spontaneous behaviour is result of the low tolerance combined with the persistence of clusters (once formed) due to aging effect and the large number of vacancies that allows the possibility of the formation of clusters at the interface.

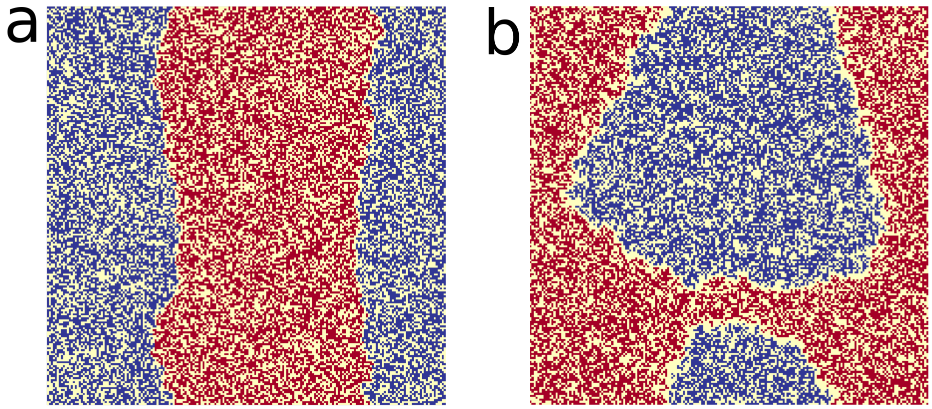

Figure 2. Snapshots of the system at the final segregated state (after 10^6 MC steps) for the Schelling model (**a**) and the version with (**b**). System size 200 x 200 with $\rho_v = 0.45$ and $T = 0.29$.

In order to quantify this vacancy cluster formation, we define a measure inspired in the segregation coefficient:

$$s_v = \frac{1}{(L^2\rho_v)^2} \sum_{\{c\}} n_c^2 \tag{1}$$

where c is the size of a vacancy cluster and n_c is the number of clusters with size c. The sample average of s_v after reaching equilibrium is called the cluster coefficient of vacancies $\langle s_v \rangle$.

The results of this measure as a function of ρ_v for a few values of T are represented in Fig.3 for the Schelling model with and without aging. We observe an increasing dependence of $\langle s_v \rangle$ with ρ_v for both models, but the effect reducing tolerance changes dramatically the behaviour for the case with aging, highlighting the vacancy cluster formation.

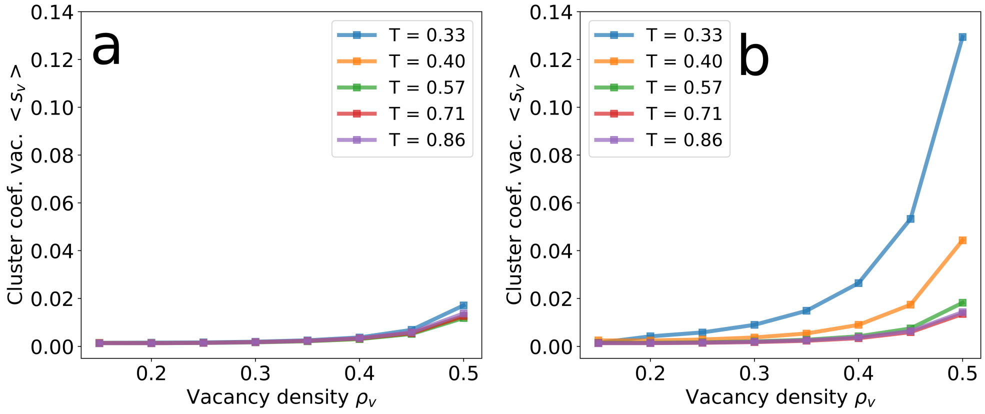

Figure 3. Cluster coefficient of vacancies as a function of the vacancy density ρ_v for the Schelling model (**a**) and the version with (**b**) for different values of the tolerance T.
